# If it ain't broke, don't fix it: evaluating the effect of increased recombination on response to selection for wheat breeding

**DOI:** 10.1093/g3journal/jkac291

**Published:** 2022-11-04

**Authors:** Ella Taagen, Katherine Jordan, Eduard Akhunov, Mark E Sorrells, Jean-Luc Jannink

**Affiliations:** Plant Breeding and Genetics Section, School of Integrative Plant Science, Cornell University, Ithaca NY 14853, USA; USDA-ARS, Hard Winter Wheat Genetics Research Unit, Manhattan, KS 66502, USA; Department of Plant Pathology, Kansas State University, Manhattan, KS 66502, USA; Department of Plant Pathology, Kansas State University, Manhattan, KS 66502, USA; Plant Breeding and Genetics Section, School of Integrative Plant Science, Cornell University, Ithaca NY 14853, USA; Plant Breeding and Genetics Section, School of Integrative Plant Science, Cornell University, Ithaca NY 14853, USA; USDA-ARS, R.W. Holley Center, Cornell University, Ithaca, NY 14853, USA

**Keywords:** wheat, meiotic recombination, deleterious variant, simulation, genomic selection

## Abstract

Meiotic recombination is a source of allelic diversity, but the low frequency and biased distribution of crossovers that occur during meiosis limits the genetic variation available to plant breeders. Simulation studies previously identified that increased recombination frequency can retain more genetic variation and drive greater genetic gains than wildtype recombination. Our study was motivated by the need to define desirable recombination intervals in regions of the genome with fewer crossovers. We hypothesized that deleterious variants, which can negatively impact phenotypes and occur at higher frequencies in low recombining regions where they are linked in repulsion with favorable loci, may offer a signal for positioning shifts of recombination distributions. Genomic selection breeding simulation models based on empirical wheat data were developed to evaluate increased recombination frequency and changing recombination distribution on response to selection. Comparing high and low values for a range of simulation parameters identified that few combinations retained greater genetic variation and fewer still achieved higher genetic gain than wildtype. More recombination was associated with loss of genomic prediction accuracy, which outweighed the benefits of disrupting repulsion linkages. Irrespective of recombination frequency or distribution and deleterious variant annotation, enhanced response to selection under increased recombination required polygenic trait architecture, high heritability, an initial scenario of more repulsion than coupling linkages, and greater than 6 cycles of genomic selection. Altogether, the outcomes of this research discourage a controlled recombination approach to genomic selection in wheat as a more efficient path to retaining genetic variation and increasing genetic gains compared with existing breeding methods.

## Introduction

Plant breeders rely on natural recombination of genetic material during meiotic segregation to generate novel allelic combinations and select favorable haplotypes. A single crossover (CO) between each homologous chromosome pair during meiosis is obligate for proper chromosome segregation. Recombination rates above this minimum can vary but are limited in most species (Henderson and Bomblies 2021). Additionally, the distribution of COs in many eukaryotes, including plants, are skewed away from the pericentromere and toward subtelomeric regions. The low frequency and uneven distribution of COs along chromosomes limits the genetic variation accessible to plant breeders, which requires working with large populations over many cycles of selection to identify new cultivars.

There are evolutionary advantages as well as costs associated with variation in recombination. The benefits of recombination are largely described by 2 models in population and evolutionary genetics: the Hill-Robertson effect and Muller’s Ratchet (Muller 1964; Hill and Robertson 1966). Recombination can disrupt repulsion linkages between favorable and deleterious loci, aiding in the efficiency of selection (i.e. the Hill–Robertson effect). In the absence of recombination, a population’s deleterious load will steadily increase and can never fall below the lowest load in the original population (i.e. Muller’s Ratchet). In regions of low recombination like the pericentromere, deleterious mutations may persist and thus are likely to become linked in repulsion with positive loci (Rodgers-Melnick *et al.* 2015; Jordan *et al.* 2018). Significantly increased recombination can come at a cost though, leading to decreased fitness by breaking up beneficial linkages and reducing fertility (Charlesworth and Barton 1996; Mieulet *et al.* 2018).

New mutations continuously arise in populations due to errors in DNA replication. Their fitness effects can range from lethal to beneficial. The rate of new mutations in eukaryotes is estimated to be at least 1×10^−8^/base pair/meiosis, and it is predicted that new mutations in coding regions will be deleterious in some of the environments the species inhabits (Ohta 1972, 1992; Baer *et al.* 2007). The accumulation of deleterious mutations may also be faster in allopolyploids, compared with diploids, due to the masking effect of wildtype (WT) gene copies (Conover and Wendel 2022). Artificial selection and population improvement (i.e. increased fitness) often result in inbreeding, which reduces the effective recombination rate (Moyers *et al.* 2018). The “cost of domestication” hypothesis suggests that the process of artificial selection has increased the proportion of deleterious variants in domesticated genomes compared with their wild progenitor (Lu *et al.* 2006; Moyers *et al.* 2018). This cost reduces the efficiency of selection, for example, as the likelihood of deleterious variants hitchhiking via linkage disequilibrium (LD) is increased.

Overcoming the low rate and biased positioning of COs to reduce deleterious load and increase the genetic variation accessible to breeders is a longstanding goal. Manipulation of pro- and anti-CO factors, and the use of genome-editing reagents that induce double-stranded DNA breaks or modify the epigenome at desired sites of recombination (e.g. CRISPR/Cas system), offer novel approaches for modifying CO frequency and distribution (Hayut *et al.* 2017; Corem *et al.* 2018; Fernandes *et al.* 2018; Mieulet *et al.* 2018; Serra *et al.* 2018; Underwood *et al.* 2018; Taagen *et al.* 2020). These approaches are collectively referred to as “controlled recombination” and transitioning them from a laboratory setting using model species to a breeder’s field with diverse applications may more efficiently harness the power of selection for plant breeding (Taagen *et al.* 2020). For example, mutation of anti-CO factor *recq4* orthologs in rice (*Oryza sativa* L.), pea (*Pisum sativum* L.), and tomato (*Solanum lycopersicum* L.), has been shown to increase CO frequency by 3-fold compared with WT (Mieulet *et al.* 2018). In Arabidopsis (*Arabidopsis thaliana*), increased copy-number of pro-CO factor HEI10 and mutation of *recq4a* and *reqc4b* additively led to 5- and 1.5-fold increased meiotic recombination in the chromosome arms and pericentromeric heterochromatin, respectively (Serra *et al.* 2018). Modified recombination distributions and frequencies have also been achieved by generating allotriploid hybrids in turnip (*Brassica rapa* L.), which reported a remarkable 20-fold increased recombination rate in the pericentromere (Pelé *et al.* 2017). All together, these methods may open the door to exciting applications for breeding, including rapid fine-mapping of genes, facilitating reintroduction of genetic variance at sites of selective sweeps, introgression of diverse alleles from wild crop relatives, and maintenance of genetic variation during genomic selection (GS) (Tam *et al.* 2011; Sadhu *et al.* 2016; Presting 2018; Rey *et al.* 2018; Taagen *et al.* 2020).

While there are a variety of methods under development to introduce controlled recombination to a crop species, determining where in the genome to implement controlled recombination is essential to enhancing breeding efficiency and genetic gain. At present, controlled recombination for inbred and hybrid crops, as well as livestock breeding pipelines has only been tested with simulation models (Battagin *et al.* 2016; Bernardo 2017; Gonen *et al.* 2017; Brandariz and Bernardo 2019; Johnsson *et al.* 2019; Ru and Bernardo 2019, 2020; Tourrette *et al.* 2019; Oyetunde and Bernardo 2020). The consensus from simulations across different species and traits is that applying controlled recombination could double genetic gains. However, it is notable that few simulations to date have factored in a cost of novel technology adoption and feasibility of multiplex genome editing. For example, under independent segregation of chromosomes into gametes, the likelihood of generating an individual with 2 desired site-specific COs on all 21 wheat chromosomes is on the magnitude of 1/2^21^. In addition, many of these simulations rely on estimated marker effects to predict ideal CO intervals that produce the greatest marker effect sum, which limits the analysis to identifying regions where historical recombination has made quantitative trait loci (QTL) effects apparent (Bernardo 2017; Brandariz and Bernardo 2019; Ru and Bernardo 2019, 2020; Tourrette *et al.* 2019; Oyetunde and Bernardo 2020). In regions of low recombination where deleterious mutations are more likely to become linked in repulsion that repulsion hides their deleterious effects. Using approaches to identify deleterious alleles, such as variant effect prediction in coding regions or evolutionary conservation, offers distinct strengths for identifying potentially advantageous controlled recombination targets in plant breeding (Rodgers-Melnick *et al.* 2015; Kono *et al.* 2018, 2019; Johnsson *et al.* 2019).

Many putative deleterious alleles have been identified in pericentromeric areas and shifting the distribution of recombination toward the pericentromere could unlock novel haplotypes and ultimately inform better targets for site-specific controlled recombination approaches. While these technologies remain under research and development, initial reports on them prompted us to test the value of recombination in regions of the genome where we suspect it has long been suppressed. We hypothesized that deleterious variants, which can occur more frequently in low recombining regions and have significant negative effects on phenotype, may offer a signal for evaluation of shifting recombination distributions.

We used empirical genotype data and deleterious variant annotations from wheat (*Triticum aestivum* L.) to simulate a GS breeding program and identified the parameter space in which increased recombination maintained genetic diversity and increased genetic gain. Previous evaluations of the recombination landscape in this diverse wheat population reported that over 75% of the recombination events fell within 10% of the distal end of chromosomes, and a higher density of putative deleterious variants were detected in the pericentromere versus distal arms of chromosomes (Jordan *et al.* 2018). We evaluated the impact of several simulation parameters on population improvement, including the number of QTL per chromosome, heritability, the recombination frequency and distribution, whether the QTL were annotated as deleterious variants, initial scenarios of coupling vs repulsion between QTL, and finally the relationship matrix used for estimated marker effects. Altogether, our findings highlight the challenging path to realizing benefits from increased recombination for a GS wheat breeding program and consider the value of adopting this technology.

## Materials and methods

**Fig. 1.**
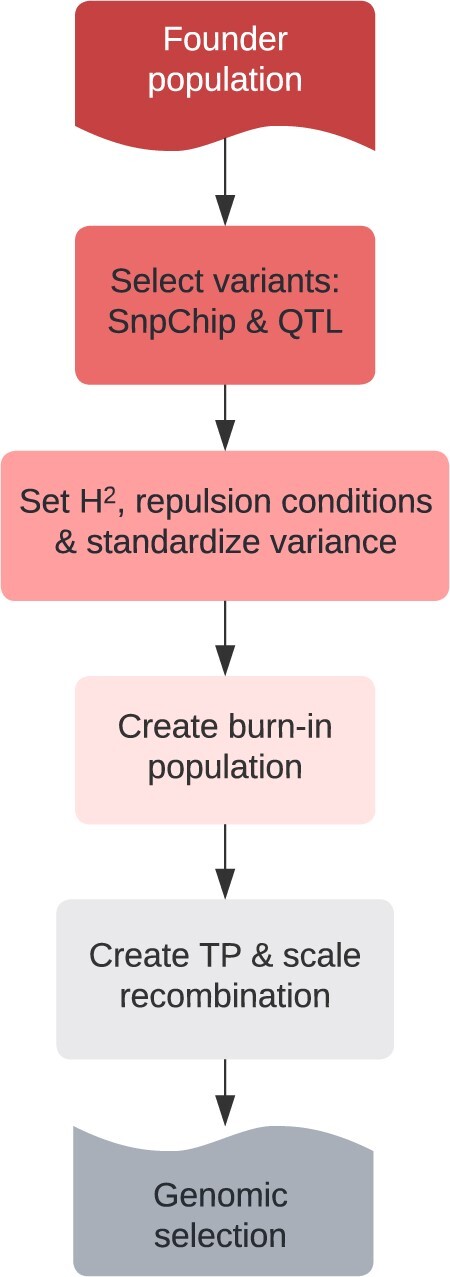
Each simulation replicate begins with a founder population, either the full set of 26 wheat accessions or PI_220431 and PI_185715 for a biparental population (see *Founder SNP, linkage map data, and population structure*). From observed variants among these founders, marker and QTL variants for simulation are selected (see *Simulation parameter: SnpChip*, *Simulation parameter: QTL*, and *Simulation parameter: deleterious variant annotations*). Simulation conditions are set (heritability and LD), and the variance is standardized to 1 (see *Simulation parameter: coupling and repulsion*). The population is burned in using cycles of phenotypic selection, the variance is again standardized to 1, and a TP is generated (see *Breeding and selection scheme*). Finally, the recombination is left unchanged or scaled up by 2- or 20-fold across the low recombining region, or across the entire chromosome (see *Simulation parameter: recombination*). GS is then simulated (see *Breeding and selection scheme*).

We used the programming language R and the breeding program simulation tool AlphaSimR to evaluate the effects of increased frequency and shifting distribution of recombination on a range of population improvement scenarios following a basic scheme (Fig. 1) (R Core Team 2020; Gaynor *et al.* 2021). The raw data, scripts for the simulations, and supplementary results are available on https://github.com/etaagen/dissertation_chapter_4 (last accessed 11/08/22).

### Founder SNP, linkage map data, and population structure

We used a published dataset of 29 genetically and geographically diverse wheat accessions for our simulations, previously selected to develop a nested association mapping population (Jordan *et al.* 2018). The high-density SNP data for the founder lines was generated using the wheat exome capture assay and mapped to the W7984 reference genome and genetic map (Chapman *et al.* 2015). We retrieved SNP and indel data (Supplementary Tables 2 and 3) from http://wheagenomics.plantpath.ksu.edu/nam and removed variants with more than 60% missing genotypes and annotations of unknown chromosomes. The remaining missing genotype calls were imputed with R/qtl2 imputation sim_geno() and monomorphic SNPs were removed (Broman *et al.* 2019).

The 29 founders were fully inbred, and we designated genotypes as “1” and “0” for the major and minor allele, respectively. A principal component (PC) analysis of the founders identified that the first PC explained 8% of the total variance and was associated with the minor allele count (Supplementary figure file path Supplementary_2/plots_S2.1). There were 3 outlier lines (*Cltr_15134*, *Cltr_11223*, and *PI_366716*) that drove the very high observed rate of coupling among minor alleles, and we removed them from the founder population (see *Simulation parameter: coupling and repulsion*). After this final data filtering step and reevaluating major and minor alleles, there were 26 founder lines, 352,804 total SNPs, 134,803 SNPs on the A genome, 181,012 SNPs on the B genome, and 36,989 SNPs on the D genome.

We also considered a biparental simulation scheme, where we selected the 2 most divergent parents from the 26 founders, *PI_220431* and *PI_185715*, whose genotypes were designated as “1” and “0,” respectively. The biparental population had 130,127 total SNPs, 61,387 on the A genome, 53,471 on the B genome, and 15,269 on the D genome. The full set of 26 lines and the biparental population served as founders for their respective simulation replicate draws.

We use “SNP” to refer to any variant in the founder population, “marker” to refer to variants assigned to the SnpChip (i.e. these generate observed genotypes in the AlphaSimR simulation), and “QTL” to refer to variants assigned as causal loci. We use the term recombination to refer to the meiotic process where a double-stranded DNA break is repaired via homologous recombination, resulting in a CO.

### Simulation parameter: SnpChip

Each replicate of the simulation (see Fig. 1) began by randomly sampling SNPs from each cM bin to serve as the markers on the SnpChip. When markers shared the same genetic map position, we jittered them by 0.00001 M. The chromosome length and marker density averaged 1.22 M and 1200 SNPs per chromosome for both the full founder and biparental population approaches.

### Simulation parameter: QTL

The QTL effects in AlphaSimR inform the genetic value, which we used to simulate a trait phenotype. All effects were additive (no dominance or epistasis). Two approaches for assigning QTL were considered, either randomly (R) or if we categorized the QTL as potentially harboring a deleterious variant (DV) based on the SnpEff annotation (see *Simulation parameter: deleterious variant annotations*). Per chromosome, we simulated either 2 (simple oligogenic trait) or 200 (complex polygenic trait) QTL, and there was no overlap between the SnpChip and QTL. We considered traits with heritability values of 0.2 and 0.8. Relationships among individuals were calculated using genome-wide (GW) marker scores from the SnpChip. We also evaluated a setting in which relationships were calculated using the scores of the causal variants (CV) at the QTL. This approach did not require an SnpChip as it used the QTL directly to estimate marker effects and is reflective of perfect LD between the markers and QTL (de Los Campos *et al.* 2013).

### Simulation parameter: deleterious variant annotations

**Fig. 2.**
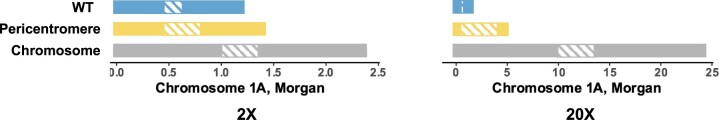
WT genetic map comparison with Pericentromere and Chromosome genetic map types with 2× (left column) and 20× (right column) increased recombination. The *X*-axis is the position along chromosome 1A in Morgans. The slash patterned lines represent the 0.2 M (WT) low recombination regions spanning the centromere on each chromosome that was designated for increased recombination in the Pericentromere map type. See Supplementary figure file path Supplementary_2/figure_S2.2.pdf for genetic maps of all 21 chromosomes.

Annotation of deleterious variants in the founder population from exome capture (coding sequence) data were previously published using the SNPeffect program (De Baets *et al.* 2012; Jordan *et al.* 2018). We considered SNPs with the “high” SnpEff putative impact criterion as well as the “nonsynonymous coding” effect to be potentially deleterious variants for DV QTL (see http://pcingola.github.io/SnpEff/). These variants were found to be more frequent in lower recombining regions, which we defined as a 0.2-M bin spanning the centromere on each chromosome (Fig. 2) (Jordan *et al.* 2018). Under the biparental population approach there was a 25% loss in SNP density and 59% loss in potentially deleterious SnpEff variants across the lower recombining regions, compared with the full 26 founder set. Given this reduction in polymorphic sites, the impact of increased recombination on DV QTL was only evaluated in the full founder population.

We interpreted the SnpEff annotation as an imperfect approximation for impact on yield. This decision was motivated by yield being a highly complex trait that is influenced by genetic variants on every chromosome and at every stage of plant growth. While the SnpEff annotation is based on predicted impact on a protein, that protein may not impact the phenotype per se, and we varied the pool of potentially deleterious variants for DV QTL each replicate of the simulation. Each replicate sampled 90% of SNPs with the “high” SnpEff putative impact criterion and 25% of SNPs with the “nonsynonymous coding” effect (Lu *et al.* 2006). This resulted in an average pool per chromosome of 613 potentially causal SNPs for the DV QTL approach. Note that the SnpEff annotations were fewest on the group D chromosomes, which reduced the number of polygenic trait QTL on the D genome. To simplify simulation comparisons, we set the number of polygenic trait QTL to match the DV QTL number, which averaged 200 QTL per A and B genome chromosomes, and 60 QTL per D genome chromosome.

### Simulation parameter: coupling and repulsion

Recombination can be beneficial for genetic improvement if it breaks up QTL in repulsion (alleles with opposing effects coinherited on the same haplotype), but detrimental if it breaks up QTL in coupling (alleles with consistent effects coinherited on the same haplotype). We estimated the amount of repulsion in the full founder population by randomly selecting 200 QTL per chromosome (R and DV approach) 100 times and measuring the % of neighboring QTL where minor alleles were in repulsion. Given the average chromosome length was 1.22 M, neighboring QTL were assumed to be in linkage. Fewer than 33% of neighboring R QTL and 18% of neighboring DV QTL had a negative correlation, which indicated the 26 founders had an inherently high degree of coupling (i.e. major alleles were primarily coinherited on the same haplotype). The 5 coupling and repulsion scenarios presented below are relative to this baseline coupling in the founder population.

In AlphaSimR the additive effects of each QTL are sampled from a standard normal distribution and the magnitude of the effects is scaled to achieve a user specified genetic variance. As we set the full founder genotypes to “1” and “0” for the major and minor allele, respectively, we could use the QTL effect sign, positive or negative, to introduce different levels of coupling and repulsion between neighboring QTL. We set different levels of coupling vs repulsion linkages among the founder QTL because we were interested in the importance of starting conditions vs ongoing selection effects on the impact of increased recombination. Intuitively, we would assume the minor allele to be deleterious (because it would have been selected against during evolution). On the other hand, domestication and the transition to new agricultural environments may cause changes in the fitness effects of alleles such that an allele historically driven to low frequency by evolution may become favorable. To test the effect of increased recombination on population improvement we tested 5 different QTL effect sign distribution scenarios:

Additive effect signs are positive for all QTL (major allele favorable for all QTL).
Random 2/3 of additive effect signs are positive and 1/3 are negative for QTL.
3. Random 1/2 of additive effect signs are positive and 1/2 are negative for QTL.
1/2 of additive effect signs are positive and 1/2 are negative, alternating positive or negative for adjacent QTL.
5. Random 1/3 of additive effect signs are positive and 2/3 are negative for QTL.

The founder and the burned-in population (see *Breeding and selection scheme*) genetic variance were always standardized to 1 by dividing each QTL effect size by the square root of the population’s additive genetic variance. The first scenario creates extreme conditions of coupling between every QTL, which resulted in selection against minor alleles. The fourth scenario creates another extreme condition of repulsion between every QTL. The third scenario is random and assumes random coupling or repulsion. The remaining scenarios present moderate ratios of coupling and repulsion, and test different proportions of selecting against the minor allele. In the biparental population approach we only applied scenarios 3 and 4.

### Simulation parameter: recombination

After the population simulation parameters were set, and the burn-in population was generated, we initiated a GS scheme (see *Breeding and selection scheme*). At the start of GS, we introduced a variable that scaled the genetic map size by 2- or 20-fold, either across the entire chromosome or only in lower recombining regions compared with the WT genetic map. These map types were, respectively, named Chromosome, Pericentromere, and WT (Fig. 2). The size of the genetic map is proportional to the amount of recombination, and we accounted for CO interference with the Kosambi mapping function.

### Breeding and selection scheme

Once the simulation parameters were assigned for the full founder population, we conducted 10 cycles of phenotypic selection to burn-in the population. Each cycle consisted of 400 random crosses among 80 doubled haploid (DH) parents, with the very first crosses among the 26 founders. Each cross produced 1 F1 progeny used to create 1 DH. Phenotypic selection was used to advance 80 DHs (20% selection intensity) to the next cycle. In the tenth generation all 400 DHs were advanced and assigned to the training population (TP). For the biparental population founders, after the simulation parameters were assigned the burn-in was only 1 cycle and all 400 unselected DHs were used to generate a TP.

At this point for the full founder or biparental TP, the WT genetic map was used, or the increased recombination parameter was applied. The TP also served as the first GS candidate population. We conducted 10 cycles GS, beginning with 400 random crosses that produced 1 F_1_ progeny used to create 1 DH each, for a total of 400 DHs. The AlphaSimR function *RRBLUP()* was used to estimate breeding values in the TP, predict breeding values of the DH candidates, and select 20 DHs (5% selection intensity) to advance to the next cycle. 160 DHs (40% selection intensity) with the greatest estimated breeding value were phenotyped and added to the existing TP each cycle, and we dropped the bottom 20% of the updated TP each cycle (i.e. the cycle 10 TP consisted of 1200 DHs).

### Simulation replicates and statistical analysis

For each cycle of GS, we calculated the additive genetic variance [AlphaSimR function *varA()*] and the genetic gain (increase in performance achieved through selection as measured by the difference between the population mean genetic value at cycle x and the population mean genetic value at cycle 1) (Gaynor *et al.* 2021). We evaluated the prediction accuracy of the estimated breeding values for the DH population at each cycle of selection by calculating the correlation with their true genetic values. We measured the impact of the Bulmer effect on genetic variance in the DHs using the AlphaSimR functions *varA()* and *genicVarA()*. The *genicVarA()* function calculates the expected variance under Hardy–Weinberg and linkage equilibrium (HWE) for each QTL and reports the sum. It thus removes the impact of LD on genetic variance. As such, we calculated Bulmer effect = *varA()*/*genicVarA()/2* (the factor of 2 is because the DH population is fully inbred, whereas *genicVarA()* assumes HWE). A stronger Bulmer effect (lower values) indicates scenarios of greater repulsion linkage between QTL, as shown by Bulmer (1971). We also measured the QTL fixation ratio for small (bottom third), medium (middle third), and large effect QTL (top third), and the change in QTL allele frequency.

**Fig. 3.**
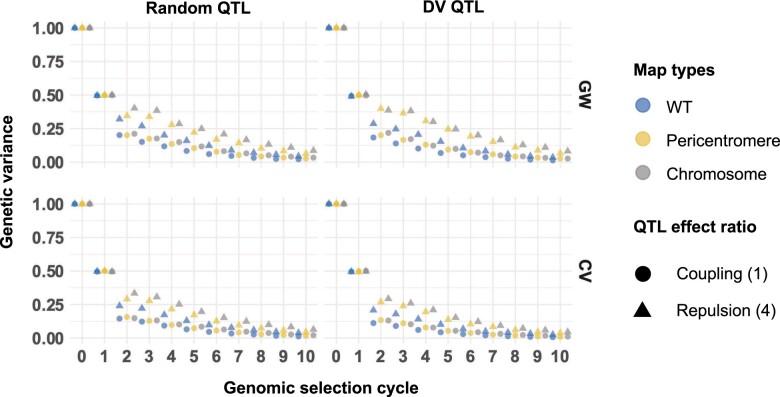
Genetic variance for the full founder population, 200 QTL per chromosome, *H*^2^ = 0.8, 20× recombination frequency. The response is measured at the end of burn-in (0 on the *x*-axis, variance standardized to 1 for all scenarios) and each cycle of GS under WT recombination (blue), pericentromere increased recombination (yellow), and full chromosome increased recombination (gray) map. QTL are assigned at random (left hand column), or QTL are assigned to deleterious variant annotations (right hand column). The rows compare whether a genomewide (GW) or causal variant relationship matrix (CV) was used to estimate marker effects. The shapes represent the levels of coupling and repulsion, circle scenario 1 (coupling) and triangle scenario 4 (repulsion) (see *Materials and methods* and Supplementary Material file path Supplementary_2/plots_S2.1 for all 5 scenarios). Each point represents the mean of 100 simulation replicates. SE were smaller than symbol sizes. The symbols are jittered horizontally for easier visualization.

**Fig. 4.**
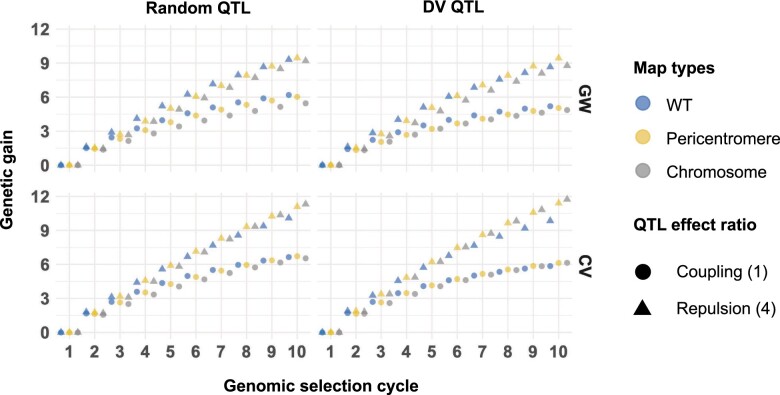
Genetic gain for the full founder population, 200 QTL per chromosome, *H*^2^ = 0.8, 20× recombination frequency. The response (mean genetic value difference between cycle X and cycle 1) is measured at each cycle of GS under WT recombination (blue), pericentromere increased recombination (yellow), and full chromosome increased recombination (gray) map. QTL are assigned at random (left hand column), or QTL are assigned to deleterious variant annotations (right hand column). The rows compare whether a genomewide (GW) or causal variant relationship matrix (CV) was used to estimate marker effects. The shapes represent the levels of coupling and repulsion, circle scenario 1 (coupling) and triangle scenario 4 (repulsion) (see *Materials and methods* and Supplementary Material file path Supplementary_2/plots_S2.1 for all 5 scenarios). Each point represents the mean of 100 simulation replicates. SE were smaller than symbol sizes. The symbols are jittered horizontally for easier visualization.

**Fig. 5.**
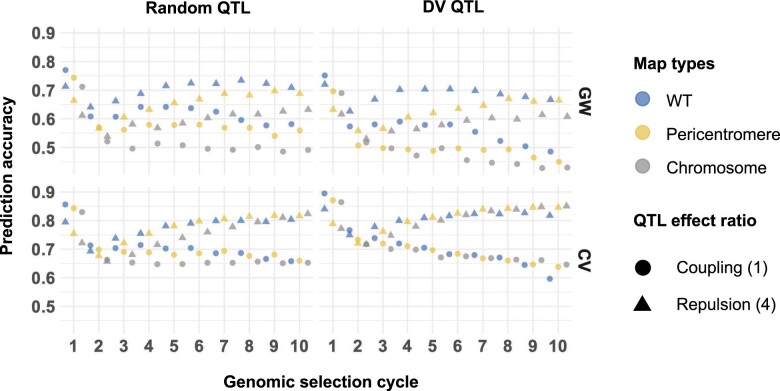
Prediction accuracy for the full founder population, 200 QTL per chromosome, *H*^2^ = 0.8, 20× recombination frequency. The response (correlation between the population’s estimated breeding values and the true genetic values) is measured at each cycle of GS under WT recombination (blue), pericentromere increased recombination (yellow), and full chromosome increased recombination (gray) map. QTL are assigned at random (left hand column), or QTL are assigned to deleterious variant annotations (right hand column). The rows compare whether a genomewide (GW) or causal variant relationship matrix (CV) was used to estimate marker effects. The shapes represent the levels of coupling and repulsion, circle scenario 1 (coupling) and triangle scenario 4 (repulsion) (see *Materials and methods* and Supplementary Material file path Supplementary_2/plots_S2.1 for all 5 scenarios). Each point represents the mean of 100 simulation replicates. SE were smaller than symbol sizes. The symbols are jittered horizontally for easier visualization.

The complete list of settings included the founder population (all 26 lines or biparental), number of QTL per chromosome (2 or 200), heritability (0.2 or 0.8), recombination frequency (2× or 20× scale), genetic map type (WT, Pericentromere, or Chromosome), QTL type (R or DV), coupling vs repulsion scenarios (1, 2, 3, 4, or 5; only 3 or 4 in biparental), and the relationship matrix (GW or CV), resulting in 672 unique simulations (Supplementary file path Supplementary_2/result_S2.1/). To account for the stochastic variation among simulations we ran 100 replicates for each setting. We fit univariate linear models with the R/lme4 package to the following response variables: genetic gain, additive genetic variance, Bulmer effect, prediction accuracy, QTL fixation, and QTL allele frequency (Bates *et al.* 2015). Separate linear models were fit at breeding cycles 6 and 10, and for the full founder and biparental simulations (Supplementary file path Supplementary_2/model_S2.1 and model_S2.2). We chose cycle 6 as it is most comparable to a traditional breeding program duration for cultivar release, and cycle 10 for a long-term comparison. The models fit fixed effects to all main effects and first-order interactions between number of QTL, heritability, recombination frequency, genetic map type, QTL type, coupling vs repulsion scenarios, relationship matrix, and allele (only for QTL fixation and QTL allele frequency: small, medium, and large effect). The replicate simulations were fit as random effects. For example, genetic gain ∼ (number of QTL + heritability + recombination frequency + genetic map type + QTL type + coupling vs repulsion scenario + relationship matrix)^2^ + (1|replicate). All models are listed in Supplementary file path Supplementary_2/model_S2.1 and model_S2.2. Analysis of variance (ANOVA) of each model and marginal means for the primary effects were evaluated. In addition, post hoc comparison of least-squares means for all significant pairwise contrasts were performed with Tukey multiple-test correction, within each model using the *R/car* and *R/emmeans* packages (Supplementary file path Supplementary_2/results_S2.2/) (Fox and Weisberg 2019; Lenth *et al.* 2019). In Figs. 3–5, we reported the mean of each measurement and did not show the SE because it was smaller than the size of the symbols on each plot.

## Results

Comparing high and low values for a range of simulation parameters (number of QTL, heritability, recombination frequency, genetic map type, QTL type, coupling vs repulsion) helped show which variables had the greatest effect on response to selection. Using values more extreme than are realistic helped us identify mechanisms that led to change in the response variables. Recombination can break genetic linkages and change the genetic variance available, but it can also diminish LD between markers and QTL. LD between markers and QTL is necessary to detect QTL. The CV relationship matrix is not affected by LD between markers and QTL, and comparisons with the GW relationship matrix results helped us disentangle the effect of LD from other variables on increased recombination. Based on evolutionary and population genetics models, and existing simulation studies, we expected that elevated recombination across the simulation conditions would slow the loss of genetic diversity and increase genetic gains over subsequent cycles of GS (Muller 1964; Hill and Robertson 1966; Gonen *et al.* 2017; Tourrette *et al.* 2019). Ultimately, we found that there was only a narrow parameter space in which increased recombination both significantly preserved genetic diversity and increased genetic gains compared with WT recombination. Unless otherwise stated, the parameters considered in the following sections are full founder population, 200 QTL per chromosome, and heritability of 0.8.

### Few simulation settings retain genetic variation and fewer still achieve higher genetic gain

We evaluated genetic variation and genetic gain responses to simulation parameters at GS cycle 6 (typical breeding program) and 10 (long term). The largest effects and interactions, by proportion of variance explained, contributing to these response variables were coupling vs repulsion, the number of QTL, heritability, and the relationship matrix (Table 1 and Supplementary file path Supplementary/model_S2.1 and model_S2.2). The smallest effects and interactions involved recombination frequency, genetic map type, and QTL type.

**Table 1.** ANOVA table proportion of variance explained summaries (%) for each genetic variance (GV) and genetic gain (GG) linear model effects at GS cycles 6 and 10.

Effect	Proportion of variance explained (%)			
	GV	GV	GG	GG
	Cycle 6	Cycle 10	Cycle 6	Cycle 10
Genetic map type	1.16	2.41	0.07	0.01
Recombination frequency	0.83	1.40	0.09	0.01
Number of QTL	50.16	62.22	9.97	29.65
Heritability	12.73	6.62	22.63	20.20
Coupling vs repulsion scenario	9.44	8.21	33.15	22.84
Relationship matrix	10.93	4.86	6.28	2.74
QTL type	0.79	1.10	0.14	0.30
Genetic map type: recombination frequency	0.35	0.70	0.04	0.01
Genetic map type: number of QTL	0.43	1.49	0.04	0.01
Genetic map type: heritability	0.02	0.15	0.01	0.03
Genetic map type: coupling vs repulsion scenario	0.23	0.30	0.01	0.02
Genetic map type: relationship matrix	0.10	0.17	0.07	0.07
Genetic map type: QTL type	0.04	0.08	0.01	0.01
Recombination frequency: number of QTL	0.24	0.66	0.02	1.13E−03
Recombination frequency: heritability	3.63E−04	0.04	3.66E−03	0.01
Recombination frequency: coupling vs repulsion scenario	0.17	0.17	0.02	0.02
Recombination frequency: relationship matrix	0.08	0.10	0.05	0.05
Recombination frequency: QTL type	0.01	0.01	9.26E−04	3.76E−03
Number of QTL: heritability	0.07	0.58	9.80	14.13
Number of QTL: coupling vs repulsion scenario	6.59	6.35	15.33	8.63
Number of QTL: relationship matrix	1.47	0.06	0.24	0.05
Number of QTL: QTL type	0.49	0.95	1.05E−03	0.08
Heritability: coupling vs repulsion scenario	1.11	0.47	0.68	0.52
Heritability: relationship matrix	1.43	0.62	0.06	0.02
Heritability: QTL type	0.04	0.02	0.02	0.03
Coupling vs repulsion scenario: relationship matrix	1.06	0.23	1.16	0.48
Coupling vs repulsion scenario: QTL type	4.87E−03	0.02	0.10	0.07
Relationship matrix: QTL type	0.03	0.03	0.01	0.01

Linear models for each GV and GG response variable effect were response variable ∼ (genetic map type + recombination frequency + number of QTL + heritability + coupling vs repulsion scenario + relationship matrix + QTL type)^2^ + (1|replicate).

In the initial F1 and DH lines, there were 1 or 2 COs per chromosome per meiosis in our WT genetic map, 2 or 3 COs in our 2× larger maps, and 14 or 15 COs in our 20× larger maps (Supplementary file path Supplementary_2/script_S2.1.R). Evaluation of 2× and 20× recombination rates compared with WT identified that 2× change in recombination rarely retained greater genetic variation, and did not increase genetic gains (Supplementary file path Supplementary_2/plots_S2.1). For example, at cycle 6 both the Chromosome and Pericentromere map type marginal mean genetic variances were at most 12% greater than WT. This trend remained at cycle 10. This small increase in genetic variance did not translate to significantly different genetic gains compared with WT. We only observed 2× recombination generate a difference in genetic variation and greater genetic gains compared with WT at cycle 10 when QTL were initially in repulsion (scenarios 3–5) and using the CV relationship matrix (Supplementary file path Supplementary_2/plots_S2.1). The same observations held for 2× recombination was present in the biparental population (Supplementary file path Supplementary_2/plots_S2.2).

Under 20× greater recombination we observed multiple simulation parameters that retained more genetic variance and had higher genetic gains than WT (Figs. 3 and 4). Similar to the previous parameters, however, increased genetic variance did not always translate to greater genetic gains. For example, at cycle 6 both the Chromosome and Pericentromere map type with 20× increased recombination had marginal mean genetic variances that were at least 30% greater than WT. This trend was even stronger at cycle 10. Yet the marginal mean genetic gains at cycle 10 were nearly equivalent for WT and Pericentromere map type, and 2.5% less for the Chromosome map type. Comparison across all marginal mean genetic gains at cycle 10 with 20× recombination identified that the only simulation settings where both the Chromosome and Pericentromere map type outperformed WT recombination were when the CV relationship matrix was used. The GW and CV relationship matrix comparisons highlighted that gain under higher recombination (i.e. >20 M) suffered from LD decay between markers and QTL leading to loss of prediction accuracy (see Fig. 5 and *Changing recombination frequency or distribution is more efficient when QTL locations are known*). Note too that even when using the CV relationship matrix, prediction accuracy decreased in early cycles of selection under high recombination (Supplementary file path Supplementary_2/plots_S2.1 and S2.2), before recovering by cycle 10. Since there was no LD decay between markers and QTL with the CV matrix, this accuracy decrease indicates that LD between QTL is also relevant to accuracy, an effect that cannot be mitigated even by knowing the causal variants (Fig. 5).

The most efficient response to selection compared with WT recombination was under the CV relationship matrix and 20× recombination for initially high repulsion (scenario 4). For these parameter settings at cycle 10 the Chromosome and Pericentromere map types both retained at least 37% more genetic variance than WT. This translated to 8.3% and 6.5% greater genetic gains in the Chromosome and Pericentromere map type, respectively, compared with WT recombination. A similar trend was observed in the biparental population scenario 4, but total genetic gains and % differences from WT were smaller (Supplementary file path Supplementary_2/plots_S2.2).

### Increased recombination is beneficial under repulsion scenarios and has a marginal impact under coupling scenarios

Thus far we have primarily considered initial populations with high repulsion between QTL (scenario 4), as this scenario had the greatest response to selection. We also evaluated moderate coupling and repulsion (scenarios 2 and 5, respectively), random (scenario 3), and high coupling (scenario 1) in the initial population. Given Chromosome or Pericentromere map type, scenario 4 had the greatest overall response to selection and difference from WT recombination compared with scenarios 1–3 and 5 (Supplementary file path Supplementary_2/plots_S2.1). High coupling (scenario 1) performed the worst, retaining the least variance and showing no consistent or significant difference in genetic gain compared with WT recombination. Focusing on the CV relationship matrix results at cycle 10 under moderate coupling, the marginal mean genetic gain compared with WT was at most 2% greater for Chromosome and Pericentromere map types. Under moderate repulsion the marginal mean genetic gain compared with WT was not significantly different for the Pericentromere map type, and 1.6% greater for the Chromosome map type. Under low heritability, 20× increased recombination had a negative impact on genetic gain for all scenarios except high repulsion (see *Genetic gain from increased recombination is less efficient for low heritability traits*).

We also evaluated the Bulmer effect across GS cycles (Supplementary file path Supplementary_2/plots_S2.1). Of the 5 initial coupling and repulsion scenarios, scenario 1 had the weakest Bulmer effect at each cycle of increased recombination, scenarios 2, 3, and 5 were not consistently different, and scenario 4 had the strongest Bulmer effect as it had the most to gain from recombining away from repulsion linkage. Marginal mean comparisons between Chromosome and Pericentromere map types were not significantly different for their Bulmer effect, suggesting both recombination frequency and distribution approaches are beneficial for breaking up repulsion linkages. However, the DV QTL had the strongest Bulmer effects compared with R QTL, as variants in regions of low recombination may have more repulsion linkages.

In addition, we measured the overall change of QTL allele frequency for large, medium, and small effect QTL (Supplementary file path Supplementary_2/plots_S2.1). Large effect QTL had the greatest change in allele frequency while small effects experienced little change. Under high repulsion we observed the small effect QTL negative allele frequency could increase. This phenomenon of increased deleterious variant allele frequency is known as genetic hitchhiking and can occur due to LD with targets of selection where the combined effect is net positive (Moyers *et al.* 2018). While there was no significant difference due to recombination frequency, distribution, or QTL type on the allele frequency change in the full founder population, there was a difference in the biparental population. Most notably, for scenario 4 in the full founder population only small effect QTL negative alleles hitchhiked but in the biparental population both small and medium effect QTL negative alleles hitchhiked.

### Increased recombination is not beneficial for oligogenic traits

We tested simulations with 2 and 200 QTL per chromosome to compare how oligogenic and polygenic traits respond to changes in recombination. With only 2 QTL per chromosome the QTL became fixed sooner. This often occurred before GS cycle 6, and genetic variance and gains plateaued. In fact, the rate of fixation of positive and negative QTL alleles showed no response to changes in heritability, recombination frequency, map type, QTL type, or relationship matrix, and only showed a response to the number of QTL per chromosome (Supplementary file path Supplementary_2/plots_S2.1).

### Genetic gain from increased recombination is less efficient for low heritability traits

In our simulations, we modeled additive traits with high (0.8) and low (0.2) heritability. More genetic variation was retained for 2× and 20× recombination under low heritability, but the relative genetic gain was significantly less than those under high heritability simulations (Supplementary file path Supplementary_2/plots_S2.1). For example, at cycle 10 across all simulation conditions the marginal mean genetic variances were 0.025 for low heritability and 0.015 for high heritability. And the marginal mean genetic gains were 3.6 and 5.7, respectively. Under low heritability the repulsion scenarios (scenarios 3–5) did not have significantly different genetic gains, and coupling scenario performed significantly worse than WT. The low heritability simulation settings had a 2-fold decrease in marginal mean prediction accuracy compared with high heritability. Increased recombination and low heritability may retain more genetic variation, but this did not translate to significant genetic gains compared with WT.

### Changing recombination frequency or distribution is more efficient when QTL are well annotated

The Chromosome (Chr) map type increased the WT recombination rate without changing the distribution, whereas the Pericentromere map type increased the WT recombination rate only in regions previously identified to have suppressed recombination (Fig. 2) (Jordan *et al.* 2018). We expected that irrespective of QTL type, the Chromosome map type would perform better than Pericentromere map type because the overall genetic map is larger. We also expected the Pericentromere map type to perform better under the DV approach, compared with the R approach because the DV QTL are primarily found in the pericentromere.

Across all simulation conditions, comparison of the marginal mean genetic gain at cycle 10 for WT, Pericentromere, and Chromosome map type revealed no significant difference (4.66, 4.68, and 4.63, respectively). Under our comparison of QTL type, the R QTL had a 6.6% higher marginal mean genetic gain than DV QTL (4.8 and 4.5, respectively). As previously noted, a significant difference between map types under 20× recombination was associated with polygenic traits, high heritability, and high repulsion. Narrowing our focus to analyzing the effect of map type under these conditions at cycle 10, we recognized that the relationship matrix played significant role in the genetic gain irrespective of map or QTL type (Figs. 3 and 4). Across these simulation settings the marginal mean genetic gain for the GW relationship matrix was 19% less than the CV relationship matrix genetic gain.

Taking these observations into account, we compared the effect of map type and QTL type for 20× recombination at cycle 10 with a polygenic trait, high heritability, and high repulsion. Under the GW relationship matrix, map types performed equivalently with R QTL. The Pericentromere map type performed the best with DV QTL, while Chromosome map type performed the same as WT (Fig. 4). Under the CV relationship matrix, Chromosome map type always performed slightly better than Pericentromere map type, and 12.2% better than WT with R QTL and 19.2% better than WT with DV QTL. While the Chromosome map type was largest, the LD decay between SNP and QTL under the GW relationship matrix neutralized the benefits of increased recombination breaking repulsion linkages. The DV QTL marginal mean genetic gains were generally lower than R QTL, but under the CV relationship matrix they were higher than R QTL raw values and significantly better than WT for both Pericentromere and Chromosome map types. If the precise locations of QTL are known, the genetic map size and the distribution of QTL may have comparable response to selection. Note that we only evaluated DV QTL in the full founder population because the biparental population had too few SNPs and SnpEff annotations in the pericentromere.

### Prediction accuracy is lower under increased recombination

Begin by considering the GW relationship matrix for comparing the prediction accuracy across simulation parameters, as the CV relationship matrix is reflective of perfect LD between the markers and QTL (Fig. 5). The marginal mean prediction accuracy at cycle 6 was 0.45 for WT recombination, 0.43 for Pericentromere map type, and 0.41 for Chromosome map type. A similar trend was observed at cycle 10. As noted previously, LD diminished between the markers and QTL for larger genetic maps, leading to lower prediction accuracy (Fig. 5). Under the CV relationship matrix, by cycle 10 the larger genetic maps had similar or improved prediction accuracy compared with WT recombination.

## Discussion

For the majority of simulation parameter scenarios studied, increased recombination did not have a significant impact on genetic gain compared with WT recombination after 10 cycles of GS. Testing high and low values of simulation parameters were important for dissecting which combinations did have an impact on translating increased recombination to greater genetic gains.

### The variance generated from increased recombination may not increase genetic gain

We observed tradeoffs in genetic gain under increased recombination due to the genetic variation generated from breaking repulsion linkages, and the variation retained from reduced prediction accuracy. Repulsion vs coupling, the relationship matrix, the number of QTL, and heritability had some of the largest effects on our measurements of genetic variance and genetic gain under increased recombination.

The effect of repulsion was most apparent in the 2× increased recombination simulations, where only scenario 4 outperformed WT recombination. This represents an extreme level of repulsion unlikely to arise in an elite breeding program but demonstrated that populations with repulsion stand to gain the most from increased recombination. It also highlights that for controlled recombination technologies to be adopted by a breeding program, they will need to achieve greater than 2× increased recombination. While this has been accomplished in Arabidopsis and some crop species, the mechanisms and response (i.e. plant viability and recombination frequency achieved) are not always conserved (Mieulet *et al.* 2018).

Under 20× increased recombination the scenarios with QTL initially in coupling phase occasionally performed worse than WT, and likely suffered from disrupted blocks of positive alleles. Scenario 4 still performed the best at cycle 6 and 10 under 20× recombination, which suggests that the benefits of increased recombination may depend on initial population conditions more so than conditions that arise inherently due to selection. Similar observations about the positive and negative response to increased recombination in repulsion and coupling scenarios, respectively, have been observed but methods to evaluate repulsion linkages in a breeding population are slow and not common practice in modern breeding (Jinks 1981; Tourrette *et al.* 2019).

When we turned our attention to 20× increased recombination, 2 competing effects became clear. First, increased recombination can break repulsion or coupling linkages, changing the genetic variance available and driving genetic gain, and second, it can diminish the LD between markers and QTL necessary for genomic prediction. For example, the relationship between increased recombination on moderate repulsion, random, and coupling starting scenarios and higher genetic gain was primarily evident with the CV relationship matrix. With the GW relationship matrix, the Chromosome map type generally performed worse than WT irrespective of initial repulsion scenarios. This observation was associated with LD decay between the markers and QTL (data not shown), which led to lower prediction accuracy than WT. The poor performance of Chromosome map type under the GW relationship matrix was not consistent with previous studies, perhaps due to a difference in methodology (Battagin *et al.* 2016; Gonen *et al.* 2017; Tourrette *et al.* 2019). To realize gains from higher recombination frequencies, i.e. 20× larger genetic maps, knowledge of the locations of QTL would be beneficial, as would increasing the marker density beyond the 1,200 markers per wheat chromosome used here.

Our designation of the major and minor allele as “1” and “0,” respectively, may have contributed to plateauing genetic gains in the coupling scenarios as the initial positive QTL allele frequencies were >0.5. For scenarios 3–5 under the DV QTL approach, at least half of the causal variants’ negative allele was the major allele, which is likely an overestimate of the population’s genetic load. Using an evolutionary conservation approach such as a Genomic Evolutionary Rate Profiling score to designate the preferred vs deleterious allele for the SnpEff variants would be more accurate for characterizing the population’s genetic load, but it is not clear if this would have lessened the baseline coupling in the founder population. Notably, comparison between the R and DV QTL approach starting conditions identified little variation in allele frequency across simulation replicates (Supplementary Material file path Supplementary_2/plots_S2.1).

We learned that increased recombination simulations with polygenic traits (200 QTL per chromosome) performed better than oligogenic traits (2 QTL per chromosome) due to slower rates of QTL fixation. There was no difference in response variables from WT recombination across the oligogenic trait simulation parameters. For the polygenic trait simulations, allele fixation occurred sooner for WT compared with increased recombination only for the biparental founder population, likely due to higher initial LD. The trend that genetic gain increased with more QTL had previously been observed by other simulation studies that increased recombination frequency (Battagin *et al.* 2016; Tourrette *et al.* 2019). When there are many QTL the loss of genetic variance is associated with the Bulmer effect, and when there are few QTL, the loss is associated with allele frequencies more quickly approaching fixation. Consequently, generally increasing the frequency or shifting the distribution of recombination may not be appropriate for oligogenic trait architecture. For example, controlled recombination simulations that use genome editing to target COs to specific intervals along each chromosome, rather than modifying the overall CO rate and distribution across many generations, may be suitable for oligogenic traits (Bernardo 2017; Brandariz and Bernardo 2019; Ru and Bernardo 2019, 2020).

In our study heritability also mediated the translation of increased recombination to greater genetic gains. The polygenic trait and low heritability simulation settings are comparable to the genetic architecture of yield. The marginal mean genetic gain of a polygenic trait at cycle 10 with low heritability for the Chromosome and Pericentromere map type were actually less than WT. Our results suggest that increased recombination is not a promising strategy for low heritability quantitative traits, e.g. yield improvement. This is different from previous controlled recombination simulation findings in rice and turnip, where the response to increased recombination under 0.8 and 0.2 heritability was very similar (Tourrette *et al.* 2019). Differences in our results for low heritability traits from existing studies may be explained by variation in methodology.

### The efficiency of increased recombination may depend on knowledge of QTL locations

Previous simulations have shown that significantly more recombination in low recombining regions increased the efficiency of selection (Gonen *et al.* 2017; Tourrette *et al.* 2019). However, when marker effects are less sensitive to repulsion linkages in regions of low recombination, how accurately can simulations evaluate the effect of increased recombination? We developed the Chromosome and Pericentromere map types, in combination with the R and DV QTL approaches to test the value of recombination in regions of the genome where historical recombination had not made QTL effects apparent. Note that this approach required a large and diverse founder population (i.e. compared with the biparental) to have sufficient marker density and SnpEff annotations to designate DV QTL.

Contrary to our original expectations, we found that the map type and QTL type had some of the smallest effects on genetic variation and genetic gain under increased recombination. Generally, the R QTL performed better than the DV QTL because they are more evenly distributed. However, when the QTL positions are known (CV relationship matrix) the DV QTL performed better irrespective of the increased map type. Indeed, enhancing recombination in regions where causal variants are concentrated is more likely to aid in selection (Gonen *et al.* 2017).

While revealing currently inaccessible genetic diversity with more recombination in the pericentromere is an exciting prospect, our simulations with data from real wheat populations indicate it is most beneficial to know the location of causal variants. This information will not be available for many traits in a typical breeding program today, but with advances in genome editing, sequencing, and deleterious variant annotation, identification or prediction of variants that underlie quantitative traits in the coming decades may increase (Ramstein and Buckler 2022).

### Feasibility of increased recombination for wheat GS breeding programs

A primary assumption that we made during our simulations was that increasing the frequency and shifting the distribution of recombination in wheat is biologically feasible. Assuming a controlled recombination method is successfully applied to wheat, our results are markedly less promising than previous simulations with comparable parameters, which have reported upwards of 34% greater gains compared with WT recombination (Tourrette *et al.* 2019). While we are not sure why increased recombination in our study had a lower response to selection than in Tourrette *et al.* (2019), responses in the latter were not uniform (i.e. between turnip vs rice). Working with wheat, our specific founder population structure, as well as R packages used to generate controlled recombination simulation parameters, could be responsible for differences in our results.

Many of the simulation parameters tested in this study (e.g. the 5 coupling and repulsion scenarios) were included because they helped to diagnose the reason for limited response to selection under increased recombination. One persistent limitation was the accuracy of GS under increased recombination, which required retraining our model at each cycle of selection. We recognize that this would be an added cost for a breeding program. A larger and more diverse population than our 26 founder lines would also likely require increasing the marker density. In addition, a differential response in genetic gain was not always detected by GS cycle 6, hence we ran our simulations through cycle 10. However, the results at cycle 6 are more representative of the time to deliver a cultivar given traditional breeding methods, and the rate of technology advancement. The time to introduce the technology to a breeding program (e.g. tissue culture methods) is another cycle of generations to consider.

The cost and resources required for adopting a genome-editing mediated technology may be prohibitive for wheat breeding programs. Our simulations showed that the benefits of increased recombination were only realized if the recombination rate was 20-fold greater than WT for many generations. However, too much recombination can cause segregation problems and decrease fertility (Pelé *et al.* 2017; Mieulet *et al.* 2018). Additionally, requiring many generations of selection may not be appropriate for all trait architectures or outweigh the benefits of traditional plant breeding methods.

### Conclusion

Given the potential applications of controlled recombination to future breeding programs, this study was motivated by the need to define desirable recombination intervals in regions of the genome where historically very few COs have been detected. Ultimately our comparison of the recombination frequency and distribution, as well as the QTL annotation to better understand the impact of increased recombination had little impact on response to selection. Increased recombination had a greater influence on selection under initial scenarios of repulsion linkage, polygenic trait architecture, and high heritability. We also showed that the increased genetic variation generated from more recombination may be associated with loss of genomic prediction accuracy (GW relationship matrix) rather than broken repulsion linkages (CV relationship matrix), which narrows the conditions in which controlled recombination may produce greater genetic gains. Collectively, the outcomes of this research challenge whether a controlled recombination application to GS in wheat offers a more efficient path to retaining genetic variation and increasing genetic gains compared with existing breeding methods.

## Data Availability

All the data referenced in this manuscript, supplementary files, custom functions, and scripts for reproducing results are publicly available at: https://github.com/etaagen/dissertation_chapter_4.
